# Rethinking Incarcerated Women's Leisure as Subjected to Coercive and Normative Prison Missions

**DOI:** 10.3389/fspor.2020.588775

**Published:** 2020-11-23

**Authors:** Alexis Marcoux Rouleau

**Affiliations:** School of Criminology, University of Montreal, Montreal, QC, Canada

**Keywords:** leisure, sports, arts, programs, prison, women, normative, coercive

## Abstract

Leisure is commonly understood as contributing to well-being; this is especially appealing when considering multiply marginalized populations such as incarcerated women. However, leisure is not impervious to cooptation by less benevolent social processes. In this conceptual analysis, I argue that incarcerated women's leisure must be rethought as a component of its environment and by extension, as subjected to coercive and normative prison missions. After broadly delineating incarcerated women's leisure, I determine that some characteristics of leisure can be compatible with these prison missions. I then examine individual, organizational, and social benefits and issues with leisure in women's prisons. I link these practices to reduced coerciveness and increased normativity. I conclude by suggesting that ensuring incarcerated people's well-being through leisure is not in itself an end, but a means to achieve prison's coercive and normative ends. I discuss implications for scholars, practitioners, and advocates.

## Introduction

Criminalized women tend to be multiply marginalized—poor, racialized, single parents, survivors of physical, and sexual violence, struggling with mental or physical health problems and addictions (Balfour and Comack, 2014). Once incarcerated, deprivations inherent to the setting engender embodied and affective suffering among this population (Chamberlen, 2016). This baseline of suffering is especially worrying in the context of the worldwide coronavirus pandemic. Indeed, as prisons lock down, concerns of human rights violations have been entwined with reported suspensions of “non-essential” prison activities including family visits, programs, and leisure (see compilation by Rubin, 2020). Such activities are, however, essential to women's coping and well-being in prison (Davila Figueroa, 2011).

From a human rights or social justice perspective, these suspensions can be dismaying. However, leisure's status as a right in prison has often been contested; leisure may instead be a privilege (Walakafra-Wills, 1983; Todd, 1995; Lee, 1996; Hensley et al., 2003; Lippke, 2003; Ambrose and Rosky, 2013; Lucas et al., 2019). If leisure is a privilege rather than a right, it can easily be denied by surveillance staff or suspended by prison administrators for internal or external motives. Further, leisure is both an individual and social phenomenon which cannot be understood as separate from the rest of social life (Rojek, 1995). Prisons are highly regulated and relatively sealed institutions (Vacheret and Lemire, 2007); it follows that attempts to understand leisure in prison should tie this phenomenon to its particular sociological setting. The question at hand thus is not “why cancel leisure if it can offer some comfort in these trying times,” but “how is leisure part of its environment in the first place?” In this paper, I argue that leisure needs to be rethought as a component of prison and as subjected to the same organizational missions.

So how does leisure fit within prison's missions? What does this entail for incarcerated women? After broadly delineating leisure in women's prisons, I examine whether leisure *could* fit by tying some of its characteristics to Canadian prisons' coercive and normative missions (Vacheret and Lemire, 2007). I then review incarcerated women's leisure practices—what it *actually does, for whom*. I link benefits and issues at individual, organizational, and societal levels to coercive and normative prison missions. In closing I discuss implications for researchers and for practitioners and advocates.

Multiple articles review the state of knowledge around yoga, sports, or arts-based programs in prison and most of the literature emphasizes how leisure contributes to incarcerated people's well-being (Finio, 1986; Cheliotis, 2014; Auty et al., 2017; Martinez-Merino et al., 2017; Woods et al., 2017). However, incarcerated women's leisure experiences are more complex than this literature suggests due to this population's trauma experiences as well as leisure's potential to reflect oppressive social structures and enact social control (Yuen et al., 2012). The current paper contributes to this body of knowledge by considering *all* types of leisure in prison, identifying benefits as well as issues, and tying these to prison missions. This provides a clearer picture of leisure as a component of its environment. In that sense, I bring together English and French empirical, theoretical, and gray literature spanning leisure and prison studies. Because leisure in women's prisons remains an emerging area of inquiry, some work centering men is included to provide organizational insights.

## Broadly Delineating Incarcerated Women's Leisure

It is important to identify what counts as leisure in women's prisons before attempting to rethink what this concept entails. Delineating leisure in broad strokes leaves space for recognizing patterns and commonalities across all leisure, which in turn helps make sense of leisure as a component of its environment.

Although leisure is often presumed to be positive or beneficial, a broader delineation may better account for the complexity and diversity of incarcerated women's experiences (Yuen et al., 2012). I thus consider positive and negative leisure, as well as what falls between these poles. Some leisure can be understood as *positive recreation* (Yuen and Pedlar, 2009). Indeed, Indigenous women's ceremonies in prison—the sweat lodge, annual Pow Wow, drum songs, and conversations with Elders—are experienced as leisure in that they foster re-creation, healing, empowerment, and reparative justice (Yuen and Pedlar, 2009; Yuen, 2011). As for incarcerated women in general, a range of activities can be included in leisure due to their positive effects: sports tournaments, active outdoor or passive indoor recreation, card games, movies, music, reading, telephoning family, holiday activities, and writing (Davila Figueroa, 2011). Due to positive effects on incarcerated women and despite common assumptions, even work, educational, and religious programs are experienced as recreation in the prison context (Davila Figueroa, 2011). Beyond recreation, some leisure can be *negative reproduction* because it enforces harmful norms (Yuen and Pedlar, 2009; details in Societal Issues section). It is reasonable to assume that some experiences rest in the *middle ground* between positive recreation and negative reproduction: leisure with less pronounced effects, with mixed positive and negative effects, or with positive effects only in some cases. Take access to cigarettes and tobacco in prison (Tewksbury and Mustaine, 2005). Incarcerated women could experience smoking as leisure due to its short-term soothing effect and the pleasant companionship of fellow smokers; the negative long-term effects of smoking and the nuisance for non-smokers remain acknowledged. Accepting a middle ground in leisure means that an activity like smoking is not exaggeratedly framed as empowering or as socially destructive.

Types of leisure can then be deduced from such a broad delineation. *Physical leisure* covers sports and physical activities like weight training (see Martinez-Merino et al., 2017; Woods et al., 2017). *Artistic leisure* includes writing, arts, crafting, theater, choir, and other creative endeavors (see Finio, 1986; Merriam, 1998; Leeder and Wimmer, 2007; Johnson, 2008; Cohen, 2009, 2019; Nugent and Loucks, 2011; Frigon, 2015; Ridha, 2018; Dewey et al., 2019; Lucas et al., 2019). Indigenous ceremonies, spiritual, or religious practices in prison are grouped as *cultural or spiritual leisure* (see Desaulniers Turgeon, 2010; Duwe, 2017; Snodgrass, 2019). *Relational leisure* includes support groups and citizen-detainee circles (see Twaddle et al., 2007; Pedlar et al., 2008, 2018; Fortune et al., 2010; Yuen et al., 2012). The above types are not mutually exclusive: for instance dance is physical and artistic, whereas yoga is physical, artistic, and spiritual (see Frigon and Jenny, 2009; Frigon, 2010, 2014, 2019; Jenny and Frigon, 2012; Bilderbeck et al., 2014; Auty et al., 2017; Bartels et al., 2019; Middleton et al., 2019; Rousseau et al., 2019). *Programs* include parenting, philosophy, nutrition, and high school courses; work and vocational training; clinical or therapeutic programs focused on intoxication or addictions (Morash et al., 1994; Batchelder and Pippert, 2002; Pollack, 2009, 2016; Williams and Walker, 2009; Firth et al., 2015; McCall, 2016; Coulombe, 2017; Crittenden and Koons-Witt, 2017; Duwe, 2017; Zhao et al., 2019). Animal programs such as canine training or zootherapy are also included here (Strimple, 2003; Smith, 2019; Wesely, 2019). Finally, most studies focus on *group leisure* whether it is managed by detained people or by staff and service providers. Reading is the only form of *solitary leisure* discussed in the literature (Sweeney, 2010; Davila Figueroa, 2011; Arford, 2013).

## Leisure's Anticipated Compatibility With Prison Missions

Prisons can be understood as more or less coercive or normative organizations (Vacheret and Lemire, 2007). Coercive organizations aim to maintain internal order through control and incentive systems for example; normative organizations transmit social norms through programming and rely on more humane detention conditions to achieve this goal (Vacheret and Lemire, 2007). In Canada, incarceration initially removed freedom in an effort to isolate, deprive, inflict suffering, and foster penitence (Vacheret, 2013). Although these coercive elements persist, criticisms have led to the inclusion of normative missions. Since the 1960s, rehabilitation efforts have relied on re-educating detained people to live within the boundaries of the law once released. Since the 1990s a new, overarching goal has emerged: protecting society. This is achieved by controlling specific populations and through social reintegration (Vacheret, 2013). These dual means of protecting society can be understood as a compromise between coercive and normative ends (Vacheret and Lemire, 2007). Indeed, professionals now rely on actuarial tools such as the Risk-Needs-Responsivity (RNR) model to assess whether criminalized people pose a risk for society and which needs/risk factors should be targeted in clinical interventions. Through this model, the responsibility to become a conventional citizen has shifted to the individual whose time spent in prison is purportedly maximized to change their so-called antisocial personality (Vacheret, 2013).

Can or should leisure fit within prison's missions? Yes, according to theorists and even media. Within a total institution such as a prison, every aspect of daily life including play occurs in the same place and under the same authority: participation is coerced and tightly regimented to comply with the institution's official missions (Goffman, 1968). Prisons aim to discipline, punish, and normalize individuals, which manifests through a control of activities: time penetrates the body and must remain maximally filled and useful so as to increase the productivity of the whole (Foucault, 1975). Even the harshest theories of punishment are compatible with at least minimal recreation and entertainment (Lippke, 2003). One study has also shown written medias' insistence that women's leisure subscribe to prison's coercive and normative missions, rather than be frivolous or trivial (Pedlar et al., 2007).

How can leisure be compatible with prison's missions? First, it can be understood as keeping detained people *active* rather than idle, which in turn may be tied to the degree of coerciveness within a prison. Idleness is indeed understood as a source of vice and crime and is further discussed as a scourge for detained people, guards, and administrators (Foucault, 1975; Wiebe and Nesbitt, 2000; Batchelder and Pippert, 2002; Martin and Kaledas, 2010). Since time is experienced as unbearably long and painful in prison (Vacheret, 2013), filling it with leisure could attenuate such hardships (Batchelder and Pippert, 2002; Ambrose and Rosky, 2013). Simply staying busy through leisure could reflect the middle ground between extremely positive and extremely negative leisure.

Second, leisure allows for *agency*, which could help incarcerated people adapt to or survive more coercive environments. Indeed, the wider literature often characterizes leisure as allowing for agency, whether through freedom, autonomy, choice, discretionary power, absence of constraints from work or other obligations (Kelly, 1972; Samdahl, 1988; Iso-Ahola, 1999; Jackson and Burton, 1999; Scraton and Watson, 2016; Roberts, 2019). In prison, three studies show leisure helps detained people adjust to deprivations, constraints, stressors, or frustrations; perceived free will is key (Kratcoski and Babb, 1990; Davila Figueroa, 2011; Meek and Lewis, 2014). Others demonstrate how leisure allows detained people to make some choices, be autonomous, and even to feel somewhat free despite prison constraints (Pedlar et al., 2008; Yuen and Pedlar, 2009; Fortune et al., 2010; Sweeney, 2010; Davila Figueroa, 2011; Link and Williams, 2017). Leisure allowing for agency could qualify as positive recreation.

Third, *conventional* leisure could help satisfy normative prison missions. As opposed to deviant leisure, conventional leisure respects formal or informal norms (Stebbins, 1997; Williams, 2009) and can aim to produce socially acceptable individuals (Yuen et al., 2012). The distinction between conventional and deviant leisure is apparent within the RNR model: people who are not involved in conventional organized leisure or who “poorly” fill their time are deemed at greater risk of reoffending (Bonta and Andrews, 2016). Prisons relying on the RNR model could enforce conventional expectations through leisure provision and be viewed as positive by staff and in public opinion; women could experience conventional leisure as reproductive and negative.

Lastly, involvement in *serious* leisure such as programs could fulfill normative prison missions. Serious leisure requires a degree of effort, perseverance, and training to be appreciated and resembles a career (Stebbins, 1982). It includes volunteering, amateurism (such as quasi-professional athletes), and hobbyism (such as collectors or tinkerers). At the other end of the spectrum, casual leisure, such as watching television or socializing with a friend, is immediately pleasant and requires no special training to be appreciated (Stebbins, 1997). Many of the reviewed documents put forth goals beyond immediate pleasure by requiring work on oneself and even by preparing individuals for conventional careers once they exit prison. For instance in one study, most incarcerated women were extremely invested in sports and exercise in prison and were even in the process of obtaining academic or professional qualifications to eventually work in this field (Ozano 2008). The broad delineation of leisure also leaves space for working on oneself to achieve positive long term goals.

## Leisure Practices as Participating in Prison's Missions

### Individual Benefits and Issues

Coercive aspects of incarceration may be reduced through leisure with *physical, psychological*, and *spiritual benefits*. Physical leisure in prison has many well-documented physical benefits established by systematic reviews (Martinez-Merino et al., 2017; Woods et al., 2017). These include improving women's cardiovascular abilities and muscular functions, relationship to their bodies, and reducing cigarette intake (Ozano, 2008; Martinez-Merino et al., 2017). Some benefits specifically address issues created by incarceration. Sports help women adopt a healthier lifestyle and manage their weight to address some consequences of poor-quality prison food (Meek and Lewis, 2014). Dance workshops answer physical issues created by incarceration, such as scarred, blocked, trapped, and encumbered bodies and eyesight; this helps transform incarcerated women's bodies (Jenny and Frigon, 2012). All types of leisure present psychological benefits. Women experience solitude as a disease: access to social activities, work, and projects help keep depression at bay (Esposito, 2015). Other benefits include reduced stress, anxiety, aggression, or other negative emotions, as well as increased self-esteem, concentration, pleasure, well-being, self-reflection, relaxation, peacefulness, connection with emotions, and positive outlook on the future (Ozano, 2008; Davila Figueroa, 2011; Jenny and Frigon, 2012; Frigon, 2014; Meek and Lewis, 2014; Esposito, 2015; Auty et al., 2017; Woods et al., 2017; Bartels et al., 2019). Indigenous women's ceremonies have spiritual, but also physical and psychological benefits by fostering holistic healing (Yuen, 2011). These practices also help resist the coercive prison setting by creating a safer space and by centering harmony and balance rather than the punitive, white, Western approach to justice (Yuen and Pedlar, 2009; Yuen, 2011).

Group leisure with *relational* or *post-detention benefits* seems closer to normative missions. Indeed, citizen-detainee groups, support circles, drama therapy, and dance workshops create opportunities to connect with self and others, to develop supportive relationships with detained people and service providers, and help create relationships extending beyond incarceration (Leeder and Wimmer, 2007; Twaddle et al., 2007; Pedlar et al., 2008; Fortune et al., 2010; Davila Figueroa, 2011; Jenny and Frigon, 2012; Frigon, 2014; Meek and Lewis, 2014). Some studies also suggest that leisure may directly benefit social reinsertion or rehabilitation. Leisure functioning in educational programs is tied to readiness for returning to society, specifically through perception of freedom and motivation in leisure (Link and Williams, 2017). Skills acquired through leisure and programs could affect one's criminal identity and help one adopt a more conventional lifestyle, which in turn may reduce or prevent reoffending (Kendall, 1993; Sempé et al., 2006; Pedlar et al., 2007, 2008; Johnson, 2008; Ozano, 2008; Yuen and Pedlar, 2009; Fortune et al., 2010; Nugent and Loucks, 2011; Yuen, 2011; Meek and Lewis, 2014; Esposito, 2015; Link and Williams, 2017). However, these results must be nuanced as all of these studies occurred while individuals were still incarcerated; they thus speak to anticipated rehabilitation, reintegration, or non-recidivism.

Although the above benefits can reduce coerciveness or contribute to normative missions, issues with leisure may tip the scale toward more coerciveness. *Psychological* and *relational issues* may increase coerciveness. Rigid and mandatory addiction treatment programs were experienced as unsafe and as inhibiting women's healing (Pollack, 2009). Return to daily prison life after a dance workshop can be difficult for trauma survivors, especially when guards act in a dehumanizing manner (Frigon, 2014). Lack of women staffing a prison's fitness center was a barrier to involvement: indeed, violence survivors and women who preferred not to be around men for cultural motives had no alternatives (Meek and Lewis, 2014). Finally, the leisure-as-rehabilitation hypothesis is dubious in cases where leisure does not fulfill incarcerated people's needs or interests (McIntosh, 1986). Such leisure could reflect the negative or preoccupied approaches to planning and implementing leisure provision, which reflect more coercive missions: the goals are, respectively, to tire detained people or fill their time without consideration for their interests (Walakafra-Wills, 1983). This sets the table for organizational practices.

### Organizational Benefits and Issues

*Organizational benefits* of leisure may contribute to coerciveness. Leisure involvement does not empirically increase a prison's safety (Frey and Delaney, 1996), yet many authors insist that individual benefits such as reducing tensions and violence can or should be leveraged to monitor and manage detained people (Walakafra-Wills, 1983; Wiebe and Nesbitt, 2000; Batchelder and Pippert, 2002; Bodin et al., 2007). Fitting leisure into an incentive system could facilitate detained people's collaboration (Sempé et al., 2006; Martin and Kaledas, 2010; Ambrose and Rosky, 2013; Bilderbeck et al., 2014; Gallant et al., 2015; Brosens, 2019). One review argues that leisure is intentionally deployed to camouflage coercive missions by controlling unruly prisoners or enforcing conformity through behavior contracts and incentive systems. Despite insistence on rehabilitation outcomes, arts-in-prison programs and their evaluations are thus used “as means to a variety of latent ignoble ends, with ‘decorative justice'—the function of masking the injustices and painful nature of imprisonment behind claims of fairness, benevolence and care—chief amongst these ends” (Cheliotis, 2014, p. 16).

*Organizational issues* in access to leisure can impede prison's normative missions by affecting individual's preparedness for returning to society (Frey and Delaney, 1996). Access may be limited due to logistic issues such as material costs, program funding, service provider salaries, scheduling conflicts, restrictions to information and resource flow (Finio, 1986; Batchelder and Pippert, 2002; Sweeney, 2010; Nugent and Loucks, 2011; Louviere, 2017; Brosens, 2019). Some structural issues can also limit access: problems informing people who speak another language, are illiterate, or are newly detained; exclusion of criminally not responsible detainees; and lack of a culture encouraging involvement in prison life (Brosens, 2019). Overcrowding and understaffing in women's prisons can also limit access to leisure resources and spaces (Nugent and Loucks, 2011; Pedlar et al., 2018). Studies also find that access to leisure in prison varies based on demographics such as gender, race, age, sentence length, and type of crime (Collette-Carrière, 1983; Kratcoski and Babb, 1990; Belknap, 1996; Batchelder and Pippert, 2002; Sempé et al., 2006; Sweeney, 2010; Meek and Lewis, 2014; Crittenden and Koons-Witt, 2017; Martinez-Merino et al., 2017).

### Societal Issues

Although leisure is often characterized as encompassing freedom, choice, escape, and satisfaction, leisure opportunities may reflect oppressive social structures such as sexism, colonialism, and racism or aim to exert social control (Rojek, 1995; Yuen and Pedlar, 2009; Yuen et al., 2012). Beyond problems in access to leisure based on gender and race, introduced above, it follows that leisure in prison may rely on gendered and racialized practices to achieve or attenuate normative and coercive missions.

Historically, Canadian prisons have relied on gender, race, and class norms in programming to reform and control women (Hannah-Moffat, 2001). More recently, authors question stereotypes reflected in leisure mostly or exclusively made available to women, such as gender-responsive or parenting programs (McCall, 2016; Crittenden and Koons-Witt, 2017; Fedock and Covington, 2017; Wendt and Fraser, 2019). Cooking, cleaning, sewing, and hairdressing training are also criticized for enforcing gendered expectations and because upon release such jobs are less likely to pay well, thereby reducing women's chances of successfully reintegrating society (Morash et al., 1994; Pollack, 2009). Women's experiences and involvement in physical leisure may also be gendered, which raises the question of how aptly physical leisure can benefit individuals in reducing coerciveness or increasing normativity. Contrary to sports or weightlifting, dance is not always conceived as a legitimate means of resistance to the coercive prison setting as it expresses sensuality, femininity, and fragility (Jenny and Frigon, 2012). Some women report they would be more involved in physical leisure if available activities reflected traditional notions of femininity (Meek and Lewis, 2014). This normative, gendered potential warrants nuance. Despite hypothesizing that incarcerated women's leisure aimed to “make good girls out of bad” by normalizing behavior, one study found that relational leisure allowed women to develop friendships which in turn fostered resistance to norms (Pedlar et al., 2008, p. 25; Fortune et al., 2010).

Racial and colonial issues can also be present within leisure and either trouble or reproduce coercive and normative missions. Incarcerated men take every decision, down to their choice of leisure, by analyzing how this affects their survival and in light of their race (Richmond and Johnson, 2009). Because traditional Indigenous leisure was historically banned or limited to support the Canadian colonial project, omitting culturally relevant activities such as lacrosse or leg wrestling can be understood as punitive and in continuity with colonialism (Yuen and Pedlar, 2009). However, attempting to curb cultural leisure to prison's normative missions may defeat its spiritual purposes. Despite the spiritual benefits of traditional Indigenous leisure in prison, such practices are often distorted for political and therapeutic purposes according to one study (Desaulniers Turgeon, 2010).

## Discussion

In this paper I have tackled incarcerated women's leisure as a component of its environment. Relying on a broad delineation of leisure, which can be positive, negative, or somewhere in the middle, I have argued that its characteristics in prison should be compatible with organizational missions. Indeed, activity and agency may reduce coerciveness, whereas conventional and serious leisure may contribute to normativity. I then argued that incarcerated women's leisure practices have a range of benefits and issues: physical, psychological, spiritual, relational, post-detention, organizational, gendered, and racial. These in turn seem tied to coercive or normative prison missions.

Most of the literature I reviewed emphasized leisure's benefits especially in terms of incarcerated women's physical or psychological well-being. Problems with access to leisure could thus be interpreted as reflecting an organizational lack of emphasis on well-being. Although Canadian prisons and jails must respect fundamental human rights, ensuring well-being is not their primary mission: protecting society by controlling individuals and favoring social reinsertion is the “paramount consideration” (Corrections and Conditional Release Act, 1992, p. 3.1; see also Loi sur le système correctionnel du Québec, 2002, p. 2). In the province of Quebec, institutional lack of emphasis on well-being is evidenced by ombudsman reports condemning affronts on incarcerated women's basic needs: lack of proper heating in the winter, clean water, and access to the yard (Protecteur du citoyen, 2017, 2019). This makes sense considering the framework used in this paper. Indeed, within the normative prison, humane detention conditions are simply a means an end: transmitting social norms (Vacheret and Lemire, 2007). More coercive prisons are not explicitly concerned with humaneness (Vacheret and Lemire, 2007) although as discussed above, individual benefits of leisure may be repurposed as incentives for compliance. As such, I contend that favoring incarcerated people's well-being through leisure only occurs insofar as this benefits the organization's missions, for instance maintaining internal order or protecting society by producing conventional individuals. I suggest that this would explain why leisure in prison has been or remains suspended in the coronavirus pandemic context.

The argument put forth in this paper remains conceptual and warrants empirical investigation. More studies explicitly considering leisure as rooted in its environment are needed, perhaps in light of prison missions beyond those discussed here (Vacheret and Lemire, 2007; Vacheret, 2013) or within other total institutions such as psychiatric hospitals (Goffman, 1968). The normative prison leisure hypothesis could be strengthened through studies measuring outcomes of in-prison leisure among individuals who have been released. A more nuanced portrait could be achieved through qualitative and quantitative studies relating prison's missions to distinct leisure types, characteristics, and practices. Specifically examining the interplay of gendered and racial norms would also further scientific understanding of leisure's role in prison. Finally, the interactions between individual, organizational, and societal benefits and issues must be examined by considering coerciveness especially. For instance, how do individuals experience problems with access to leisure in prison? Can differential access make women feel like they are being punished? Is some leisure explicitly provided for punitive purposes?

Rethinking incarcerated women's leisure through the lens of normative and coercive prison missions also has implications for practitioners and human rights/social justice advocates. These groups may especially wish to address psychological and relational issues with women's leisure as these seem to contribute to coerciveness. However, attempts to increase access to leisure or to ensure its status as a human right in prison implies grappling with a dilemma. In order to effectively make this case, practitioners and advocates would need to demonstrate that leisure is essential for the organization and not exclusively for individuals. But is this a desirable argument? Do advocates and practitioners really want to argue for leisure producing conventional, acceptable women, or to argue for leisure which baits women into compliance and subservience? Would that not distort leisure's potential for freedom, choice, satisfaction, and empowerment? Perhaps incarcerated women should decide.

## Author Contributions

The author confirms being the sole contributor of this work and has approved it for publication.

## Conflict of Interest

The author declares that the research was conducted in the absence of any commercial or financial relationships that could be construed as a potential conflict of interest.
